# P-730. Risk Factors for Severe Influenza and Respiratory Syncytial Virus Infections in Patients with Lymphoma or Multiple Myeloma - A Seven-Year Retrospective Cohort Study

**DOI:** 10.1093/ofid/ofae631.926

**Published:** 2025-01-29

**Authors:** Tali Shafat, Daniel De-la-Rosa-Martinez, Fareed Khawaja, Ying Jiang, Amy Spallone, Marjorie Batista, Ella Ariza Heredia, Diana Vilar-Compte, Roy F Chemaly

**Affiliations:** The University of Texas MD Anderson Cancer Center, Houston, Texas; Instituto Nacional de Cancerologia, Mexico City, Distrito Federal, Mexico; The University of Texas MD Anderson Cancer Center, Houston, Texas; The University of Texas MD Anderson Cancer Center, Houston, Texas; University of Texas MD Anderson Cancer Center, Houston, Texas; Department of Infectious Diseases, AC Camargo Cancer Center, São Paulo, SP, Brazil., São Paulo, Sao Paulo, Brazil; The University of Texas MD Anderson Cancer Center, Houston, Texas; Instituto Nacional de Cancerología, Mexico City, Distrito Federal, Mexico; University of Texas MD Anderson Cancer Center, Houston, Texas

## Abstract

**Background:**

Respiratory viral infection (RVI) is a significant complication in patients with hematologic malignancies (HM). While risk factors and outcomes of severe infections have been studied in allogeneic hematopoietic cell transplant (HCT) recipients, data is limited for patients with lymphoma and multiple myeloma (MM). We investigated the risk factors and outcomes associated with severe respiratory syncytial virus (RSV) or influenza virus (IFV) infections from a large cohort of patients with lymphoma or MM.

**Figure 1:**
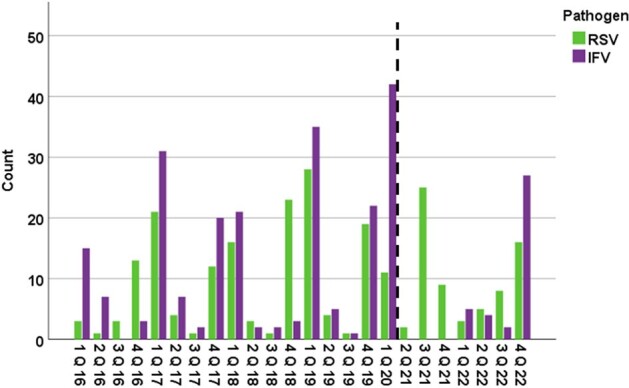
Respiratory viral infections per quarter stratified by viral pathogen. The dashed line represents the beginning of the COVID-19 era in the USA (March 2020). Abbreviations: IFV=influenza; Q=quarter; RSV= respiratory syncytial virus.

**Methods:**

We performed a retrospective study in adult patients with lymphoma or MM who were diagnosed with RSV or IFV RVIs between 2016 and 2022 and followed up to 1 year. Primary outcomes were progression to lower respiratory tract infection (LRI) and all-cause 30- and 90-day mortality.

**Figure 2:**
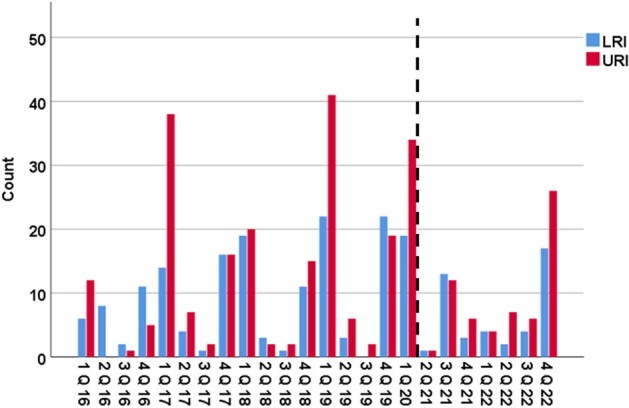
Respiratory viral infections per quarter stratified by site of infection. The dashed line represents the beginning of the COVID-19 era in the USA (March 2020). The LRI group includes patients who presented with LRI or progressed from URI to LRI. Abbreviations: LRI=lower respiratory tract infection; Q=quarter; URI= upper respiratory tract infection

**Results:**

We analyzed 440 patients with 490 consecutive viral episodes: 297 (61%) in patients with MM, and 193 (39%) with lymphoma. RVIs were secondary to RSV and IFV in 258 (52%) and 234 (48%) episodes, respectively. A decline in RVIs since the onset of the COVID-19 pandemic was noted, with an uptrend in the last quarter of 2022 (Figures 1, 2). Upon presentation, 303 (62%) patients were diagnosed with upper respiratory tract infection (URI) and 187 (38%) with LRI; while 19 patients (6%) with URI progressed to LRI. During follow-up, 57% were hospitalized, 8% were admitted to the ICU, 20 (4%) died within 30 days, and 32 (7%) within 90 days (Table 1). On multivariable analysis, RSV infection (vs. IFV), current/former smoking, recent steroid exposure, and lymphopenia or renal injury, were associated with LRI. Survival analysis revealed an association between MM (vs. lymphoma), current/former smoking, lymphopenia, and nosocomial infection and increased 30-day mortality risk, whereas LRI (vs. URI), current/former smoking, and lymphopenia were associated with 90-day mortality (Table 2).

**Table 1:**
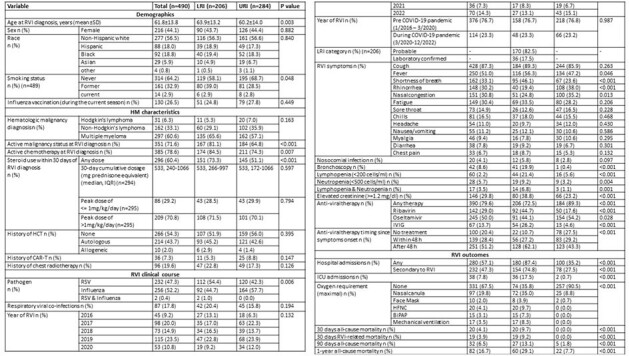
Baseline characteristics and clinical outcomes following respiratory viral infections by site of infection. Abbreviations: BiPAP= bilevel positive airway pressure; CAR-T= chimeric antigen receptor T-cell therapy; COVID-19=Coronavirus Disease 2019; HCT= hematopoietic stem cell transplantation; HFNC= high-flow nasal cannula; HM= hematologic malignancy; ICU=intensive care unit; IQR= interquartile range; IVIG= Intravenous immunoglobulin; LRI= lower respiratory tract infection; RSV= respiratory syncytial virus; RVI= respiratory virus infection; SD=standard deviation; URI= upper respiratory tract infection.

**Conclusion:**

We identified risk factors in lymphoma and MM patients with RVIs associated with substantial morbidity and mortality. Clinical parameters such as smoking status, recent steroid exposure, lymphopenia, and renal injury could potentially identify high-risk patients, thereby enabling better management strategies.

**Table 2:**
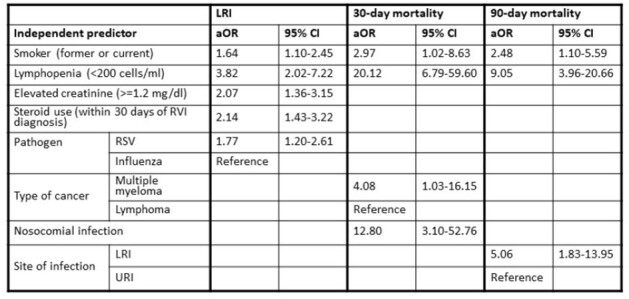
Independent predictors of LRI, 30-day and 90-day mortality by multivariable logistic regression. Abbreviations: aOR=Adjusted-Odds Ratio; LRI= lower respiratory tract infection; RSV= respiratory syncytial virus; RVI=respiratory tract infection; URI= upper respiratory tract infection; 95% CI= 95% Confidence Interval.

**Disclosures:**

**Fareed Khawaja, MBBS**, Eurofins Viracor: Grant/Research Support|Symbio: Grant/Research Support **Roy F. Chemaly, MD/MPH**, AiCuris: Advisor/Consultant|AiCuris: Grant/Research Support|Ansun Pharmaceuticals: Advisor/Consultant|Ansun Pharmaceuticals: Grant/Research Support|Astellas: Advisor/Consultant|Eurofins-Viracor: Grant/Research Support|InflaRX: Advisor/Consultant|Janssen: Advisor/Consultant|Karius: Advisor/Consultant|Karius: Grant/Research Support|Merck/MSD: Advisor/Consultant|Merck/MSD: Grant/Research Support|Moderna: Advisor/Consultant|Oxford Immunotec: Advisor/Consultant|Oxford Immunotec: Grant/Research Support|Roche/Genentech: Advisor/Consultant|Roche/Genentech: Grant/Research Support|Shinogi: Advisor/Consultant|Takeda: Advisor/Consultant|Takeda: Grant/Research Support|Tether: Advisor/Consultant

